# The mortality burden of cachexia or weight loss in patients with colorectal or pancreatic cancer: A systematic literature review

**DOI:** 10.1002/jcsm.13510

**Published:** 2024-08-02

**Authors:** Richard F. Dunne, Jeffrey Crawford, Karen E. Smoyer, Thomas D. McRae, Michelle I. Rossulek, James H. Revkin, Lisa C. Tarasenko, Philip D. Bonomi

**Affiliations:** ^1^ Department of Medicine and Wilmot Cancer Institute, Division of Hematology/Oncology University of Rochester Medical Center Rochester New York USA; ^2^ Duke Cancer Institute Duke University Medical Center Durham North Carolina USA; ^3^ Envision Pharma Group Fairfield Connecticut USA; ^4^ Department of Internal Medicine, Pfizer Research and Development Pfizer Inc New York New York USA; ^5^ Internal Medicine Research Unit, Pfizer Research and Development Pfizer Inc Cambridge Massachusetts USA; ^6^ Global Medical Affairs Pfizer Inc New York New York USA; ^7^ Department of Internal Medicine, Division of Hematology, Oncology and Cell Therapy Rush University Medical Center Chicago Illinois USA

**Keywords:** Cachexia, Colorectal cancer, Muscle wasting, Pancreatic cancer, Systematic literature review, Weight loss

## Abstract

Cancer‐associated cachexia is a multifactorial wasting disorder characterized by anorexia, unintentional weight loss (skeletal muscle mass with or without loss of fat mass), progressive functional impairment, and poor prognosis. This systematic literature review (SLR) examined the relationship between cachexia and survival in patients with colorectal or pancreatic cancer in recent literature. The SLR was conducted following PRISMA guidelines. Embase^®^ and PubMed were searched to identify articles published in English between 1 January 2016 and 10 October 2021 reporting survival in adults with cancer and cachexia or at risk of cachexia, defined by international consensus (IC) diagnostic criteria or a broader definition of any weight loss. Included publications were studies in ≥100 patients with colorectal or pancreatic cancer. Thirteen publications in patients with colorectal cancer and 13 with pancreatic cancer met eligibility criteria. Included studies were observational and primarily from Europe and the United States. Eleven studies (42%) reported cachexia using IC criteria and 15 (58%) reported any weight loss. An association between survival and cachexia or weight loss was assessed across studies using multivariate (*n* = 23) or univariate (*n* = 3) analyses and within each study across multiple weight loss categories. Cachexia/weight loss was associated with a statistically significantly poorer survival in at least one weight loss category in 16 of 23 studies that used multivariate analyses and in 1 of 3 studies (33%) that used univariate analyses. Of the 17 studies demonstrating a significant association, 9 were in patients with colorectal cancer and 8 were in patients with pancreatic cancer. Cachexia or weight loss was associated with significantly poorer survival in patients with colorectal or pancreatic cancer in nearly two‐thirds of the studies. The classification of weight loss varied across and within studies (multiple categories were evaluated) and may have contributed to variability. Nonetheless, awareness of cachexia and routine assessment of weight change in clinical practice in patients with colorectal or pancreatic cancer could help inform prognosis and influence early disease management strategies.

## Introduction

Cachexia is a multifactorial metabolic syndrome of wasting characterized by anorexia, unintentional weight loss, decreased skeletal muscle mass, and progressive functional impairment that cannot be reversed by the provision of nutritional support.^1^, ^2^, ^3^ Systemic inflammation may also play a significant role in cachexia.^4^, ^5^ Cachexia is prevalent among patients with cancer—estimated to be approximately 30% across all cancer types^6^—and can be highly burdensome. Indeed, in patients with cancer, cachexia is considered a comorbidity,^7^ which may impact quality of life, increase adverse effects from treatment, and reduce survival.^1^, ^3^, ^8^ Multiple factors can impact cachexia prevalence estimates, including cancer type,^5^, ^6^ stage,^6^, ^9^ treatment,^1^, ^3^, ^10^ patient sex,^11^ and the presence of other comorbidities.^12^ In addition, certain cancer therapies (e.g., platinum‐based agents) have the potential to exacerbate weight loss and/or muscle wasting.^10^

Although cachexia is highly prevalent and an indicator of poor prognosis in patients with cancer, reaching consensus on a clinically meaningful definition and appropriate diagnostic criteria to identify those with, or at risk of cachexia, has been challenging. To facilitate diagnoses, a landmark international consensus (IC) definition was derived in 2011, with accompanying diagnostic criteria (weight loss >5% over the previous 6 months [in the absence of simple starvation]; or ongoing weight loss >2% and body mass index [BMI] < 20 kg/m^2^; or ongoing weight loss >2% and skeletal muscle mass loss consistent with sarcopenia). A disease classification system was published^2^ and subsequently validated in an international patient sample with advanced cancer.^13^ However, studies frequently use either more narrow definitions (>5% weight loss only) or broader definitions of cachexia or weight loss, including cachexia of any definition or that is undefined, weight loss >5% but without specifying a time period or for a time period other than 6 months, or any weight loss. This heterogeneity in definition can hinder meaningful comparison of data.

Colorectal cancer (CRC) and pancreatic cancer are both leading causes of cancer‐related death.^14^, ^15^ Both cancers of the gastrointestinal tract are associated with a risk of developing cachexia.^6^ Shibata et al.^16^ reported a 50.7% cumulative incidence of cancer cachexia (at 24 weeks after starting first‐line treatment) in patients with advanced CRC, while Hendifar et al.^17^ and Latenstein et al.^18^ reported that 60–70% of patients with pancreatic cancer presented with cachexia at diagnosis. Given this high prevalence of cachexia in patients with colorectal or pancreatic cancers, a comprehensive understanding of the impact of cachexia on survival may help to improve disease management strategies, coordinated patient care, and prognosis.

## Objectives

The objectives of this systematic literature review (SLR) were two‐fold: (1) to assess the prevalence of cachexia or weight loss in adult patients with colorectal or pancreatic cancer and (2) to assess the relationship between cachexia or weight loss and overall survival (OS) in these patients.

## Methods

### Study design and eligibility criteria

**Figure 1:**
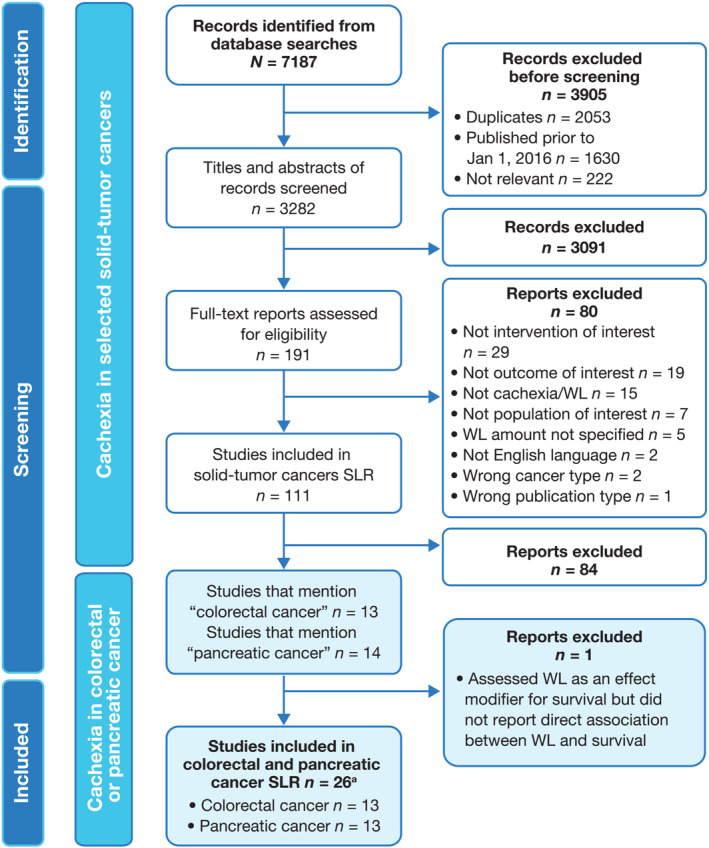
PRISMA flow diagram for identification of relevant studies for the colorectal or pancreatic cancer SLR. ^a^A total of 25 individual publications were identified, but 1 publication (Gannavarapu et al., 2018^20^) reported weight‐loss and survival data for colorectal and pancreatic cancer populations separately and was counted twice. PRISMA, Preferred Reporting Items for Systematic Reviews and Meta‐Analyses; SLR, systematic literature review; WL, weight loss. Reproduced (with modifications) from Bonomi PD et al. Mortality burden of pre‐treatment weight loss in patients with non‐small‐cell lung cancer: A systematic literature review and meta‐analysis. *Journal of Cachexia, Sarcopenia and Muscle*, 2024 Apr 22 [19] licensed under CC BY 4.0.

Literature searches identifying studies in adult patients with colorectal or pancreatic cancer and cachexia, or at risk of cachexia, were conducted as part of a broader SLR on cachexia in selected solid‐tumour cancers^19^ (Figure 1). The SLR was conducted in accordance with the Preferred Reporting Items for Systematic Reviews and Meta‐Analyses (PRISMA) 2020 statement^21^ and the PRISMA Protocol (PRISMA‐P) guidelines.^22^ The protocol was prospectively registered on January 24, 2022 (registration number: CRD42022284170) in the International Prospective Register of Systematic Reviews (PROSPERO).

Pre‐specified inclusion and exclusion criteria were determined, from which relevant studies were identified according to the study populations, interventions, comparators, outcomes, and study types (PICOS) framework (*Table* 1). For the broader SLR, adults with solid‐tumour cancers (excluding skin, oesophageal, gastric/stomach, or head and neck cancer) with cachexia, or at risk of cachexia, were eligible for inclusion. For each study, cachexia was defined according to one of two sets of criteria: (1) the IC diagnostic criteria for cachexia proposed by Fearon et al.^2^ or (2) broader criteria for cachexia or any weight loss (*Table* 2).

**Table 1:** Summary of study eligibility criteria

Parameter	Included	Excluded
Populations	Adults with solid‐tumour cancers (other than those excluded) and cachexia or at risk of cachexia, including populations identified by the international consensus criteria for cachexia or by more broad criteria for cachexia, as detailed in Table 2	Studies in paediatric populations Studies without results specific to solid‐tumour cancers Studies of patients with skin cancer Studies of patients with oesophageal, gastric/stomach, or head/neck cancers Studies without patients with cachexia (as per international consensus or broader diagnostic criteria) Patients unable to orally consume food or who are restricted to liquid nutrition
Interventions	Any or none other than those excluded	Interventions intended for weight loss Parenteral or enteral nutrition Surgery or resection
Comparators	Any or none other than those excluded	Interventions intended for weight loss Parenteral or enteral nutrition Surgery or resection
Outcomes	Overall survival or mortality in patients with cancer and cachexia (as per international consensus or broader diagnostic criteria)	Studies not reporting overall survival or mortality in patients with cancer and cachexia (as per international consensus or broader diagnostic criteria) Studies reporting weight loss as an adverse event or presenting symptom without survival or mortality data for the weight loss population
Study types^a^	Randomized or non‐randomized clinical trials Retrospective or prospective real‐world/observational studies Study types as above with ≥100 patients	Pre‐clinical, animal, and case studies, economic modelling studies (e.g., cost‐effectiveness analyses) Notes, commentaries, editorials, opinions, letters, meta‐analyses, reviews^a^ Studies with <100 patients
Other limits	Publications in English and published between 1 January 2016 and 10 October 2021 Studies in colorectal or pancreatic cancer populations	Publications not in English or published prior to 2016 Studies not in colorectal or pancreatic cancer populations

^a^ Reviews were excluded but reference lists of relevant systematic reviews were screened for primary sources. Reproduced (with modifications) from Bonomi PD et al. Mortality burden of pre‐treatment weight loss in patients with non‐small‐cell lung cancer: A systematic literature review and meta‐analysis. *Journal of Cachexia, Sarcopenia and Muscle*, 2024 Apr 22 [19] licensed under CC BY 4.0.

**Table 2:** Criteria for cancer cachexia and unintentional weight loss

**International Consensus Diagnostic Criteria for Cancer Cachexia**
Diagnostic criteria as described in Fearon et al., 2011, for patients with cancer: Weight loss >5% in the previous 6 months, OR Weight loss >2% in the previous 6 months AND one of the following: Body mass index <20.0 kg/m^2^, OR Evidence of muscle depletion (sarcopenia), such as: Appendicular skeletal muscle index determined by dual energy X‐ray absorptiometry (men <7.26 kg/m^2^; women <5.45 kg/m^2^) Mid upper‐arm muscle area determined by anthropometry (men <32 cm^2^; women <18 cm^2^) Lumbar skeletal muscle index determined by computed tomography imaging (men <55 cm^2^/m^2^; women <39 cm^2^/m^2^) Whole body fat‐free muscle mass index without bone determined by bioelectrical impedance (men <14.6 kg/m^2^; women <11.4 kg/m^2^) Absolute muscularity below the 5th percentile

**Broader Criteria for Defining Cancer Cachexia or Weight Loss**
Any one of the following, for patients with cancer: Presence of cachexia (any definition or undefined), OR Any weight loss, OR Body mass index <20.0 kg/m^2^ AND one of the following indicators of sarcopenia: Presence of sarcopenia (any definition or undefined) Any skeletal muscle mass depletion Low muscle density or any reduction in muscle density Low muscle strength^a^ Low muscle quantity or quality^a^ Poor physical performance^a^

^a^ Sarcopenia indicators as reported in the Revised European Working Group on Sarcopenia in Older People (EWGSOP2) (Cruz‐Jentoft et al., 2019^23^).

Study type and study intervention eligibility are detailed in *Table* 1. Eligible study outcomes were OS or mortality in patients with cancer and cachexia (according to the IC or broad definition, including any weight loss). Publications included in this SLR were of studies in ≥100 patients with colorectal or pancreatic cancer.

### Data sources and search strategy

Searches were performed in Embase® (via Ovid; excluding conference abstracts) and PubMed databases on 11 October 2021. A detailed search strategy for each database is presented in *Table* S1 and *Table* S2. An original search ranging from 1 January 2011 to 10 October 2021, identified a large number of publications, and so the start date was amended to 1 January 2016 to focus on the most recent data (*Figure* 1). Included studies were limited to those published in English‐language, peer‐reviewed journals between 1 January 2016 and 10 October 2021. A manual search of reference lists from publications included in the SLR and from relevant reviews was also conducted to identify any additional, relevant publications.

### Study selection

The publication selection process is presented in *Figure* 1. Initially, records from the database searches were combined, duplicates removed, and a pre‐screen was conducted by a single researcher to exclude those considered irrelevant. Records excluded at this stage were checked by a second researcher. Studies were then selected for inclusion following a two‐level screen. Firstly, two independent reviewers screened the titles and abstracts of the remaining records against the inclusion and exclusion criteria. Secondly, full‐text screening of records identified as eligible was conducted against the pre‐defined criteria, again by two independent reviewers. Screening discrepancies were discussed between reviewers and a consensus reached. For the purposes of this current SLR, a subset of studies conducted in patients with colorectal or pancreatic cancer was then identified.

### Data extraction and quality assessment

Population demographics and baseline characteristics were extracted into pre‐specified data extraction tables, alongside reported measures of survival or mortality and cachexia or weight loss. Data from observational studies and clinical trials were reported separately. In studies where both univariate and multivariate analyses were provided, only multivariate analysis results were extracted.

Extraction of data elements and quality assessment of included text were performed by one reviewer and assessed for accuracy by a second reviewer. Longitudinal studies were assessed for risk of bias using the Newcastle–Ottawa Scale (NOS) for cohort studies.^24^ A modified NOS was used for the quality assessment of cross‐sectional studies.^25^

## Results

### Study selection and critical appraisal

A total of 7187 records were identified from the database searches (*Figure* 1). Of 3282 records that underwent title and abstract screening, 191 progressed to full‐text review. Of 111 eligible publications related to cachexia in solid‐tumour cancers, 27 studies mentioned patients with colorectal (*n* = 13) or pancreatic cancer (*n* = 14). One study in patients with pancreatic cancer assessed weight loss as an effect modifier for survival but lacked an evaluation of direct association between weight loss and survival.^26^ This study was excluded and 13 studies each for colorectal and pancreatic cancer were included for further analysis (*Table* 3). One publication^20^ reported weight loss and survival data for colorectal and pancreatic cancer populations separately and was counted in both patient populations. In total, 25 publications were included in the SLR. The definition and assessment time period of weight loss/cachexia varied by study, as outlined in *Table* S3 and *Table* S4, from prior to or at diagnosis and/or during or following treatment.

**Table 3:** Summary of CRC and pancreatic cancer studies included in the SLR

Author, year	Cancer type; main treatment type	Analysis type
**CRC studies (*n* = 13)**		
Best et al., 2021	mCRC; targeted and standard chemotherapy	MVA
Gannavarapu et al., 2018	Multi‐tumour including CRC; treatment NS	MVA
Guercio et al., 2020	mCRC; FOLFIRI or modified FOLFOX6 combined with cetuximab, bevacizumab, or a combination of cetuximab and bevacizumab	MVA
Islam et al., 2020	mCRC; bevacizumab ± conventional chemotherapy	MVA
Karabulut et al., 2021	mCRC; Chemotherapy: FP, FP + oxaliplatin/FP + irinotecan or FP + oxaliplatin + irinotecan	UVA
Kocarnik et al., 2017	CRC; treatment NS	MVA
Lee et al., 2020	Stage III or high‐risk stage II colon cancer; adjuvant FOLFOX4, bevacizumab‐FOLFOX4, bevacizumab‐XELOX following curative surgery	MVA
Liu et al., 2021	mCRC; (targeted treatment) cetuximab, bevacizumab	MVA
Meyerhardt et al., 2017	Stages I–III invasive CRC; chemotherapy and radiation	MVA
Shibata et al., 2020	Advanced CRC; First‐line chemotherapy XELOX/FOLFOX/SOX ± bevacizumab, FOLFIRI/IRIS/irinotecan ± bevacizumab, capecitabine/S‐1/FL ± bevacizumab, FOLFIRI/irinotecan + cetuximab/panitumumab, FOLFOX + cetuximab/panitumumab	MVA
Silva et al., 2020	CRC; radiotherapy, surgery, chemotherapy	MVA
Vergidis et al., 2016	Stage III colon cancer; chemotherapy	MVA
Walter et al., 2016	CRC; treatment NS	MVA
**Pancreatic cancer studies (*n* = 13)**		
Arthur et al., 2016	Combined cancers (pancreatic cancer cohort); treatment type NS	MVA
Carnie et al., 2020	PDA; triplet combination, doublet combinations and monotherapy chemotherapy	MVA
Domínguez‐Muñoz et al., 2018	Pancreatic cancer; chemotherapy with pancreatic enzyme replacement therapy	UVA
Duconseil et al., 2019	BRPC or LAPC; chemotherapy, chemoradiation, surgery	MVA
Gannavarapu et al., 2018	Multi‐tumour including pancreatic; treatment NS	MVA
Hendifar et al., 2018	PDA; chemotherapy, surgery	MVA
Hue et al., 2021	PDA; mixed treatment (NS)	MVA
Latenstein et al., 2020	Pancreatic cancer; surgery, palliative chemotherapy, best supportive care	UVA
Mitsunaga et al., 2020	Advanced PDA; first‐line chemotherapy, modified FOLFIRINOX, gemcitabine monotherapy	MVA
Naumann et al., 2019^a^	Locally advanced pancreatic cancer; chemoradiotherapy	MVA
Naumann et al., 2019^b^	Locally advanced pancreatic cancer; chemoradiotherapy	MVA
Nemer et al., 2017	PDA; chemotherapy	MVA
Ramsey et al., 2019	PDA; chemotherapy	MVA

BRPC, borderline resectable pancreatic cancer; CRC, colorectal cancer; FOLFIRI, irinotecan, 5‐fluorouracil, and leucovorin; FL, 5‐fluorouracil + leucovorin; FP, fluoropyrimidine; IRIS, irinotecan plus S‐1; LAPC, locally advanced pancreatic cancer; FOLFOX, oxaliplatin, 5‐fluorouracil, and leucovorin; FOLFIRINOX, leucovorin, 5‐fluorouracil, irinotecan, oxaliplatin; mCRC, metastatic colorectal cancer; MVA, multivariate analyses; NS, not specified; PDA, pancreatic ductal adenocarcinoma; SOX, S‐1 plus oxaliplatin; UVA, univariate analyses; XELOX, oxaliplatin and capecitabine.

^a^ Naumann P, et al. Cancers (Basel) 2019;11:1655.^27^

^b^ Naumann P, et al. Cancers (Basel) 2019;11:709.^28^

All 13 of the CRC studies and 12 of the 13 pancreatic cancer studies identified were observational cohort studies and were subject to critical appraisal and quality assessment using the NOS (*Table* S5). Among the CRC studies, 12 scored 8 or 9, indicating a low risk of bias.^16^, ^20^, ^29^, ^30^, ^31^, ^32^, ^33^, ^34^, ^35^, ^36^, ^37^, ^38^ One study scored 7, indicating a medium risk.^39^ For the pancreatic cancer studies, 10 studies scored 8 or 9^17^, ^18^, ^20^, ^27^, ^28^, ^40^, ^41^, ^42^, ^43^, ^44^ and two studies scored 7.^45^, ^46^ A single cross‐sectional study, which included a pancreatic cancer population, was assessed for risk of bias using the modified NOS and scored 8, indicating a low risk of bias^47^ (*Table* S6).

### Characteristics of studies identified in the systematic literature review

#### Colorectal cancer studies

All 13 CRC studies were observational; 10 were retrospective studies,^16^, ^20^, ^29^, ^31^, ^33^, ^34^, ^35^, ^36^, ^37^, ^38^ and three prospective^30^, ^32^, ^39^ (*Table* 3). The design and subject characteristics of the included studies are detailed in *Table* S3. Most studies (5/13) were conducted in the United States or Canada.^29^, ^30^, ^31^, ^35^, ^37^ Three studies were from Asia^16^, ^33^, ^39^ two were from Germany^34^, ^38^ and one study was conducted in Brazil.^36^ One study^32^ was conducted across centers in the United States, Canada, and Australia, while one study^20^ did not report the country.

All study participants were adults with CRC (*N* = 16 296); the proportion of females ranged from 27.6%^34^ to 61.0%.^39^ Three studies reported mean baseline BMI, ranging from 27.4 to 27.8 kg/m^2^.^29^, ^31^, ^32^ Performance status (PS) was recorded in six studies, five using the Eastern Cooperative Oncology Group (ECOG) PS scale,^16^, ^30^, ^33^, ^34^, ^37^ and one using the World Health Organization (WHO) PS scale.^39^ Most patients across these studies had an ECOG or WHO PS grade of 0 or 1.

#### Pancreatic cancer studies

All 13 pancreatic cancer studies were observational; 10 were retrospective,^17^, ^20^, ^27^, ^28^, ^40^, ^42^, ^43^, ^44^, ^45^, ^46^ two were prospective^18^, ^41^ and one was a cross‐sectional study^47^ (*Table* S4). Most studies were conducted in Europe^18^, ^27^, ^28^, ^40^, ^41^, ^45^ and the United States.^17^, ^43^, ^44^, ^46^, ^47^ A single study was conducted in Japan.^42^

All study participants were adults with pancreatic cancer (*N* = 20 639), and the proportion of females ranged from 41.3%^42^ to 56.7%.^46^ Four studies reported mean baseline BMI, ranging from 23.6 to 31.2 kg/m^2^.^28^, ^41^, ^43^, ^44^ PS was reported in seven studies; six employed the ECOG PS scale,^27^, ^28^, ^40^, ^41^, ^42^, ^43^ and one used the WHO PS scale.^18^ Across all studies, most patients had an ECOG or WHO PS grade of 0 or 1.

### Assessment of cachexia and weight loss and association with survival or mortality

Twelve of the 13 CRC studies used multivariate analyses to evaluate the association between cachexia or weight loss and survival/mortality (*Table* 3). Three studies (23%) reported cachexia using IC criteria,^16^, ^20^, ^29^ while 10 studies (77%) met the broad definition of cachexia^30^, ^31^, ^32^, ^33^, ^34^, ^35^, ^36^, ^37^, ^38^, ^39^ (*Table* S3).

Eleven of the 13 pancreatic cancer studies used multivariate analyses^17^, ^20^, ^27^, ^28^, ^40^, ^41^, ^42^, ^43^, ^44^, ^46^, ^47^ (*Table* 3). Eight studies met the IC criteria for cachexia,^17^, ^18^, ^20^, ^27^, ^28^, ^42^, ^43^, ^44^ and five met the broad definition of cachexia^40^, ^41^, ^45^, ^46^, ^47^ (*Table* S4).

### Prevalence of cachexia and weight loss

**Figure 2:**
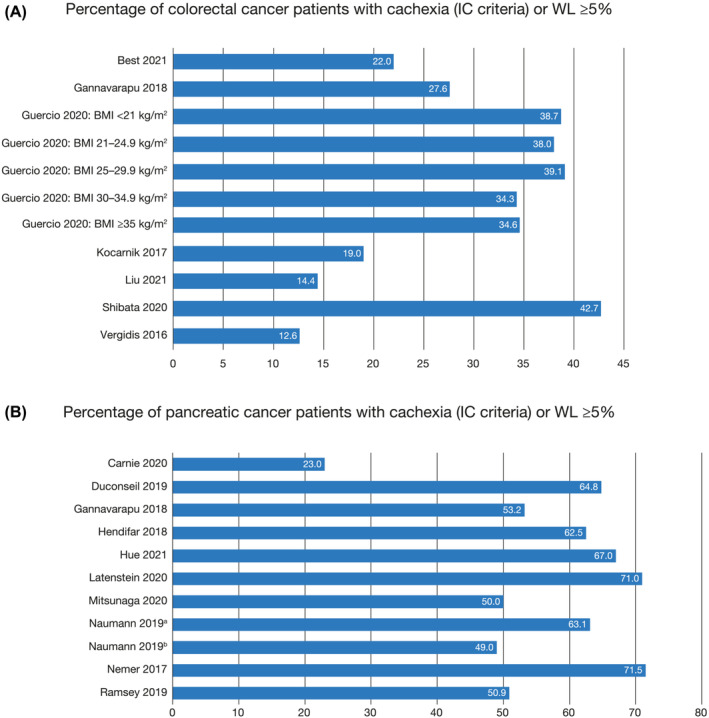
Prevalence of cachexia (IC criteria or weight loss ≥5%) in patients with (A) colorectal cancer and (B) pancreatic cancer. ^a^Naumann P, et al. Cancers (Basel) 2019;11:1655.^27^ ^b^Naumann P, et al. Cancers (Basel) 2019;11:709.^28^

The first objective of this SLR was to assess the prevalence of cachexia or weight loss in patients with colorectal or pancreatic cancer. As the SLR included studies with various definitions of cachexia or weight loss, we summarized the prevalence of patients experiencing cachexia or weight loss ≥5% to enable more consistent comparison across studies. Seven of 13 CRC studies reported prevalence (using the IC criteria or ≥5% weight loss) ranging from 12.6%^37^ to 42.7%^16^ (*Figure* 2A; *Table* S3). Eleven pancreatic cancer studies reported baseline cachexia or weight loss, ranging from 23%^40^ to 71.5%^44^ (*Figure* 2B; *Table* S4). In a US study that used ICD‐9 codes to identify cachexia in patients admitted to hospital, prevalence was 6.7% in patients with pancreatic cancer^47^ (*Table* S4). However, it should be noted that ICD‐9 codes rely on clinicians billing for cachexia and is subject to underdiagnosis.^48^

### Relationship between cachexia or weight loss, and overall survival

The second objective of the SLR was to assess the relationship between cachexia or weight loss and OS in patients with colorectal or pancreatic cancer. Overall, cachexia or weight loss was associated with statistically significantly poorer survival or greater odds of mortality in at least one weight loss or cachexia category in 16 of 23 studies (9/12 in colorectal cancer and 7/11 in pancreatic cancer) that used multivariate analyses, and in 1 (in pancreatic cancer) of 3 studies that used univariate analyses.

## Colorectal cancer studies

**Figure 3:**
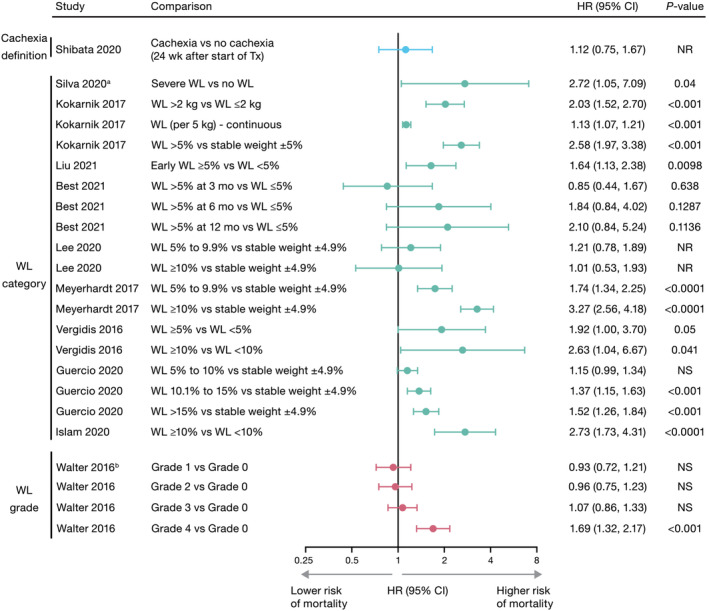
Association between cachexia or weight loss and overall survival from multivariate analyses in colorectal cancer studies. Of the 12 multivariate analyses in studies on colorectal cancer, 11 studies are shown: The remaining study (Gannavarapu et al., 2018^20^) did not report an HR, but identified a significant association between WL ≥ 5% and worse survival. Overall, nine studies using multivariate analyses showed that for at least one category assessed, cachexia or WL was associated with a statistically significant poorer survival in patients with colorectal cancer. The 1 univariate analysis (Karabulut et al., 2021^39^) showed no difference in survival for WL > 10% versus <10%. Studies have been grouped by cachexia definition, WL category, and WL grade. ^a^HR was inversed to present severe WL versus no WL (reference category). ^b^Grade 0: BMI ≥ 25 and WL < 2.5%; Grade 1: BMI 20 to <25 and WL < 2.5% or BMI ≥ 28 and WL 2.5% to <6%; Grade 2: BMI 20 to <28 and WL 2.5% to <6% or BMI ≥ 28 and WL 6% to <11%; Grade 3: BMI < 20 and WL < 6% or BMI 20 to <28 and WL 6% to <11%; Grade 4: BMI < 20 and WL ≥ 6% or BMI 20 to <22 and WL ≥ 11% or BMI 22 to <28 and WL ≥ 15%. Data are shown on a log_2_ scale. BMI, body mass index; CI, confidence interval; HR, hazard ratio; mo, months; NR, not reported; NS, not significant; Tx, treatment; wks, weeks; WL, weight loss.

CRC studies using multivariate analyses and reporting a hazard ratio (HR) and 95% confidence interval (CI) for the association between cachexia or weight loss and OS are shown in *Figure* 3. Unless specifically noted, most studies compared weight loss ≥5% (or ≥10%) as a yes/no category, so weight loss <5% (or <10%) would include smaller weight losses, stable weight, and weight gain. A single study using multivariate analysis reported the association using log‐rank *p*‐values.^20^ Overall, nine studies reported a significant association between cachexia or weight loss and poor survival. Two of these studies were prospective. Guercio et al.,^30^ reporting a prospective study in patients with metastatic colorectal cancer (mCRC), observed that greater weight loss during the 6 months prior to study entry was associated with shorter OS. Patients with weight loss of >15% or weight loss of 10.1% to 15% had greater all‐cause mortality than patients with stable weight (±4.9%) (HR: 1.52 [95% CI: 1.26–1.84] and HR: 1.37 [95% CI: 1.15–1.63], respectively). In a second prospective study that assessed the association of long‐term weight change in the 5‐year period following CRC diagnosis with long‐term survival, continuous modelling demonstrated that per 5 kg weight loss following CRC diagnosis, there was a significant association with lower OS (HR: 1.13 [95% CI: 1.07–1.21]).^32^ In a retrospective analysis of the FIRE‐3 clinical trial, Liu et al.^34^ evaluated the prognostic and predictive relevance of early weight loss (defined as body weight loss of ≥5% after 3 months of first‐line folinic acid, fluorouracil and irinotecan plus cetuximab or bevacizumab) on survival in patients with mCRC (*n* = 326). Early weight loss ≥5% was found to be an independent negative prognostic factor for OS compared with weight loss <5% (HR: 1.64 [95% CI: 1.13–2.38]; *P* = 0.0098).

Six other retrospective studies demonstrated a significant association between cachexia or weight loss and poor survival with details provided in *Figure* 3 and *Table* S3.^20^, ^31^, ^35^, ^36^, ^37^, ^38^ The retrospective study by Gannavarapu et al.^20^ in a cohort of 623 patients with primary CRC found that patients with overt weight loss (defined as meeting IC criteria for cachexia) had a shorter median OS (56.3 months; *P* < 0.001) than those with minimal weight loss (below IC criteria), or no weight loss (median OS not reached). This study did not report a hazard ratio, so is not included in *Figure* 3.

Three studies reported a non‐significant association between cachexia or weight loss and OS.^16^, ^29^, ^33^ Best et al.^29^ reported a retrospective cohort study of 226 patients with mCRC. Multivariate analysis of weight loss >5% at 3, 6, and 12 months after diagnosis of mCRC showed no significant impact on OS compared with weight loss ≤5% (3 months HR: 0.85 [95% CI: 0.44–1.67]; *P* = 0.6380; 6 months HR: 1.84 [95% CI: 0.84–4.02]; *P* = 0.1287; 12 months HR: 2.10 [95% CI: 0.84–5.24]; *P* = 0.1136).

Lee et al.^33^ retrospectively analysed data from patients (*n* = 3449) with stage III or high‐risk stage II colon cancer from the phase 3 AVANT trial, which investigated the efficacy of adding bevacizumab to standard adjuvant chemotherapy following curative resection. Weight loss ≥5% during the first 6 months of adjuvant chemotherapy was measured; however, analyses found no association with OS. However, unlike the other studies included in the SLR, this trial only enrolled patients who recently underwent surgery for colon cancer and were followed during adjuvant chemotherapy. It is common for individuals to gain weight during this period because of the amount of weight loss immediately after surgery. This could explain the low incidence (10%) of ‘cachexia’ (weight loss ≥5%) and the lack of a significant association between weight loss and OS. A retrospective study by Shibata et al.^16^ assessed the relationship between cachexia (per IC criteria) and OS in 150 patients with advanced CRC following first‐line systemic chemotherapy. OS was significantly different between patients with and without cancer cachexia within 24 weeks after starting first‐line chemotherapy (log‐rank *P* = 0.0467); median survival time for patients with and without cancer cachexia was 720 days (95% CI: 570–820) and 816 days (95% CI: 704–930), respectively. However, landmark analyses conducted at 24 weeks revealed no significant difference between groups (log‐rank *P* = 0.0823). Similarly, there was no difference in OS according to presence or absence of cancer cachexia within 12 weeks and 48 weeks of starting of chemotherapy.

## Pancreatic cancer studies

**Figure 4:**
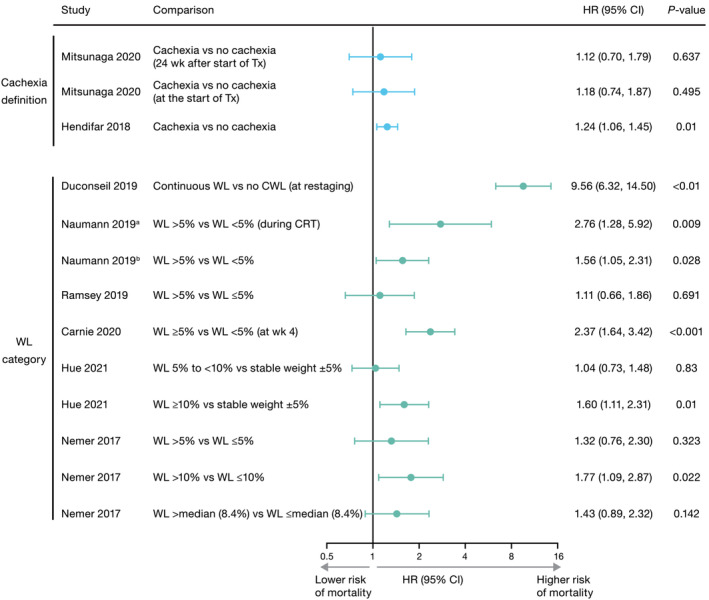
Association between cachexia or weight loss and overall survival from multivariate analyses in pancreatic cancer studies. Of the 11 multivariate analyses in studies on pancreatic cancer, nine studies are shown. The remaining two studies (Arthur et al., 2016^47^ and Gannavarapu et al., 2018^20^) did not report an HR, and both had non‐significant results. Overall, seven studies using multivariate analyses showed that for at least one category assessed, cachexia or WL was associated with a statistically significantly poorer survival for patients with pancreatic cancer. Of the two studies (Latenstein et al., 2020^18^ and Domínguez‐Muñoz et al., 2018^45^) that used univariate analyses, one (Latenstein et al., 2020^18^) demonstrated a significant association. Studies have been grouped by cachexia definition and WL category.^a^Naumann P, et al. Cancers (Basel) 2019;11:1655.^27^ ^b^Naumann P, et al. Cancers (Basel) 2019;11:709.^28^ Data are shown on a log_2_ scale. CI, confidence interval; HR, hazard ratio; CRT, chemoradiotherapy; CWL, continuous weight loss; Tx, treatment; wk, week; WL, weight loss.

Seven out of nine pancreatic cancer studies using multivariate analyses and reporting a HR and 95% CI for the association between cachexia or weight loss and OS identified a significant association (*Figure* 4). Duconseil et al.^41^ was a prospective study conducted in 454 consecutive patients diagnosed with resectable locally advanced pancreatic cancer (LAPC). In this study, continuous weight loss at restaging was associated with a significantly shorter OS (HR: 9.56 [95% CI: 6.32–14.50]; *P* < 0.01). Two retrospective studies using the IC definition for cachexia were conducted in the same cohort of patients with unresectable LAPC treated with neoadjuvant chemoradiation therapy (CRT). Naumann et al.^27^ assessed body composition and laboratory markers for cancer cachexia before and after neoadjuvant CRT in 141 patients and reported that weight loss of >5% during CRT remained independently associated with a shorter OS compared with weight loss <5% (HR: 2.76 [95% CI: 1.28–5.92]; *P* = 0.009). In a multivariate analysis of 147 patients, Naumann et al.^28^ reported that average OS was significantly shorter among patients with weight loss of >5% compared with patients who had weight loss <5% at first follow‐up (HR: 1.56 [95% CI: 1.05–2.31]; *P* = 0.028). An additional four retrospective studies reported a significant association between cachexia or weight loss and OS^17^, ^40^, ^44^, ^46^ (*Figure* 4, *Table* S4).

Four pancreatic cancer studies found no significant association between cachexia or weight loss and survival or mortality. One cross‐sectional study by Arthur et al.^47^ that assessed the risk of inpatient death for patients with pancreatic cancer, reported higher risk in patients with cachexia (broad definition following ICD‐9 diagnostic criteria) compared with those without cachexia, but the difference was not significant (OR: 1.16 [95% CI: 0.93–1.45]). However, this study uniquely assessed the risk of inpatient death, not mortality risk in general or OS. In addition, ICD‐9 diagnostic codes for cachexia might also have been under‐reported in hospitalized patients. A study by Gannavarapu et al.^20^ evaluated the prevalence and survival impact of cancer‐associated weight loss using IC criteria in patients with lung or GI cancers, including pancreatic cancer, but did not report associated HRs. Though weight loss was associated with OS when examining all cancer types (*P* < 0.001), a significant association was not observed for pancreatic cancer (*P* = 0.66), which made up only 8% of the cohort. Ramsey et al.^43^ conducted a retrospective analysis of 136 patients with biopsy‐proven PDAC. In this study, weight loss >5% was not associated with a shorter survival (HR: 1.11 [95% CI: 0.66–1.86]; *P* = 0.691) compared with weight loss ≤5%. Lastly, in another retrospective study by Mitsunaga et al.,^42^ which included 150 patients who underwent first‐line chemotherapy following diagnosis of advanced PDAC, OS was not significantly different between patients with and without follow‐up cachexia (IC definition), regardless of whether cachexia was identified at treatment initiation or within 12, 24, or 48 weeks.

Two pancreatic cancer studies evaluated the association between cachexia or weight loss and survival using univariate analyses,^18^, ^45^ one of which^18^ reported a significant association between weight loss and shorter survival.

## Discussion

This SLR was conducted to assess the prevalence of cachexia or weight loss in adult patients with colorectal or pancreatic cancer and to evaluate the association between OS and cachexia or weight loss, either before or after their diagnosis of cancer. Twenty‐five publications in patients with colorectal (*n* = 13) or pancreatic cancer (*n* = 13) were analysed, including Gannavarapu et al.,^20^ which reported outcomes for colorectal and pancreatic cancer populations separately and was therefore counted in both cancer types. The publications represent patient populations in multiple geographic locations and clinical settings, with cancer cachexia or weight loss defined by IC criteria or broader diagnostic criteria. Overall, the results show that cachexia or weight loss is prevalent in these patient populations and is associated with statistically significantly poorer survival or greater odds of mortality in at least one weight‐loss or cachexia category in 16 of 23 studies (9/12 in CRC and 7/11 in pancreatic cancer) that used multivariate analyses, and in one (in pancreatic cancer) of three studies that used univariate analyses. However, there was substantial heterogeneity across studies, and there were no notable differences in cancer stage or other factors (with the exception of Lee et al.^33^ who examined early‐stage patients who underwent recent colectomy, unlike the other included studies) that might explain why some studies (*n* = 9) did not find worse survival with cachexia. Small sample sizes or under‐diagnosis of cachexia may have contributed to the lack of significance in some studies, specifically the study utilizing ICD‐9 codes recorded at hospital admission, which reported a very low prevalence of cachexia.^47^

### Key findings and recommendations based on the SLR

Of the eligible studies included in this SLR, most were observational and primarily from Europe and the United States. Prevalence of cancer cachexia or weight loss ≥5% ranged broadly from 12.6%^37^ to 42.7%^16^ in patients with CRC and from 23.0%^40^ to 71.5%^44^ in patients with pancreatic cancer. Although these wide‐ranging data likely reflect that cachexia and weight loss vary among cancer stages and some studies included both early and advanced cancers, they also demonstrate the variation in the definition, measurement, and assessment time period of cachexia across studies. Nonetheless, they do serve to highlight the high prevalence of cachexia in these patient populations.

Fewer than half of the studies (*n* = 11 [42%]) reported cachexia using IC criteria; the remaining studies (*n* = 15 [58%]) reported cachexia/any weight loss using non‐IC/broader criteria. Despite the heterogeneity in classification of cachexia and weight loss across and within studies, this SLR highlights the potential prognostic value of cachexia diagnosis in patients with colorectal or pancreatic cancer. Overall, the association found in many of these studies between significant weight loss, either using the IC criteria of 5% or higher, is alarming because cancer‐associated weight loss is commonly seen in clinics, but it is also often overlooked. This may be due to low awareness of cachexia and its harms, or due to the lack of standard FDA‐approved therapies to treat cachexia.^3^ Physicians report difficulty in diagnosing cancer cachexia; for example, in a survey of US community oncologists, the prevalence of cancer cachexia, particularly in patients with lung cancer, was grossly underestimated.^49^ Early recognition of cachexia has become increasingly important. In a recent observational cohort study, patients with oesophagogastric cancer and cachexia who were referred for dietetic counselling early in their disease course experienced less weight loss than those who did not undergo early dietetic counselling.^50^ Additionally, though standard therapies for all patients with cachexia are lacking, oncologists can tailor treatments to control symptoms like nausea, poor appetite, and exocrine insufficiency that can lead to or exacerbate cachexia. Lastly, there are numerous pharmacologic agents and treatment strategies that are currently in active clinical trials.^51^ With the anticipation of newer and better therapeutic options, it will be essential to increase awareness of the cachexia syndrome and the need to address it, given its impact on survival. Existing clinical practice guidelines on cancer cachexia in adult patients include standardized, regular screening of at‐risk patients using validated tools (including regular assessment of weight and nutritional and metabolic status), provision of nutritional advice and education, and psychological and palliative support; such support should be implemented now, as it will become more important as the field advances.^1^, ^3^

### Strengths and limitations of the systematic literature review

This SLR addresses an important knowledge gap in the association of cachexia or weight loss with survival/mortality in patients with colorectal or pancreatic cancer. The systematic approach and quality appraisal of the included studies is a key strength; however, the study is not without limitations. Firstly, potential selection and publication bias may have resulted from including only those studies published in English language between 1 January 2016 and 10 October 2021, and indexed in Embase or PubMed databases. Secondly, heterogeneity across the included studies was large, including differences in country, population, treatment received, and study design. As noted previously, the classification of cachexia and weight loss varied across and within studies (with multiple categories evaluated). Some aspects of cachexia (e.g., imaging‐based body composition or inflammatory markers) were seldom included in the definition of cachexia in the majority of identified studies. Of note, most of the available evidence on the association between cachexia or weight loss and survival is from retrospective, observational studies. In addition, some studies described weight loss before cancer diagnosis, whereas others reported weight loss after or during cancer treatment. Lastly, not all studies used multivariate analyses. A need exists for well‐designed prospective cohort studies with standardized, clearly defined diagnostic criteria for cachexia or unintentional weight loss to better understand the mortality burden of cachexia in the growing cancer population.

## Conclusions

Cachexia or weight loss is prevalent in patients with colorectal or pancreatic cancers. Overall, cachexia or weight loss was associated with statistically significantly poorer survival or greater odds of mortality in at least one weight loss or cachexia category in nearly two‐thirds of studies assessed, highlighting the importance of consensus definitions to aid diagnosis. Increased awareness of cachexia and recording weight measurements in colorectal and pancreatic cancer patients at every clinic visit are needed. These data have important implications for prognosis, individual patient management, and clinical trials.

## Funding

This study was sponsored by Pfizer Inc, New York, NY, USA.

## Conflict of interest

RFD: Honoraria (Helsinn Healthcare, Merck & Co., Exelixis, Toray Industries). PDB: Honoraria (Pfizer, Helsinn Healthcare, Roche Genentech). JC: Consulting/advisory role and honoraria (Actimed Therapeutics, AVEO, Enzychem Lifesciences, Faraday Pharmaceuticals, G1 Therapeutics, Merck, Partner Therapeutics, Pfizer, Sandoz, BIO Alta, Seagen); research funding (Helsinn Healthcare, AstraZeneca, Pfizer [to institution]). KES: Employment (Envision Pharma Group); Stock or other ownership (Envision Pharma Group). MIR and LCT: Employment (Pfizer); Stock or other ownership (Pfizer). TDM and JHR were employees of Pfizer at the time of study execution and are Pfizer shareholders.

## Supporting information


**Table S1.** Embase^®^ search strategy.
**Table S2.** PubMed search strategy.
**Table S3.** Design and subject characteristics of colorectal cancer studies identified during the SLR (*n* = 13).
**Table S4.** Design and subject characteristics of pancreatic cancer studies identified during the SLR (*n* = 13).
**Table S5.** Quality assessment of observational studies using the Newcastle–Ottawa Scale.
**Table S6.** Quality assessment of cross‐sectional studies using the modified Newcastle–Ottawa Scale.
