# Supplementary material for: The mortality burden of cachexia or weight loss in patients with colorectal or pancreatic cancer: A systematic literature review
Source: J Cachexia Sarcopenia Muscle. 2024 Aug 2;15(5):1628–40. doi: 10.1002/jcsm.13510 (PMC11446707; doi:10.1002/jcsm.13510)
Supplement: Supplementary file 1 — Table S1. Embase® search strategy. Table S2. PubMed search strategy. Table S3. Design and subject characteristics of colorectal cancer studies identified during the SLR (n = 13). Table S4. Design and subject characteristics of pancreatic cancer studies identified during the SLR (n = 13). Table S5. Quality assessment of observational studies using the Newcastle–Ottawa Scale. Table S6. Quality assessment of cross‐sectional studies using the modified Newcastle–Ottawa Scale. [file JCSM-15-1628-s001.docx]

SUPPORTING INFORMATION

# The mortality burden of cachexia in patients with colorectal or pancreatic cancer: A systematic literature review

Richard F. Dunne^1^, Jeffrey Crawford^2^, Karen E. Smoyer^3^, Thomas D. McRae^4^*^†^*, Michelle I. Rossulek^5^, James H. Revkin^5^*^†^*, Lisa C. Tarasenko^6^ & Philip D. Bonomi^7^

*^1^Department of Medicine and Wilmot Cancer Institute, Division of Hematology/Oncology, University of Rochester Medical Center, Rochester, NY, USA; ^2^Duke Cancer Institute, Duke University Medical Center, Durham, NC, USA; ^3^Envision Pharma Group, Fairfield, CT, USA; ^4^Department of Internal Medicine, Pfizer Research and Development, Pfizer Inc, New York, NY, USA; ^5^Internal Medicine Research Unit, Pfizer Research and Development, Pfizer Inc, Cambridge, MA, USA; ^6^Global Medical Affairs, Pfizer Inc, New York, NY, USA; ^7^Department of Internal Medicine, Division of Hematology, Oncology and Cell Therapy, Rush University Medical Center, Chicago, IL, USA*

*^†^Affiliation at the time the review was conducted*

Correspondence to: Richard F. Dunne MD, [Richard_Dunne@URMC.Rochester.edu](mailto:Richard_Dunne@URMC.Rochester.edu)

**Table S1.** Embase® search strategy.

| **Step** | **Search string** |
| --- | --- |
| 1 | exp cachexia/ or exp sarcopenia/ |
| 2 | (cachexia or sarcop?nia).ti,ab. |
| 3 | (emaciation or wasting or weight loss or weight reduction).ti,ab |
| 4 | 1 or 2 or 3 |
| 5 | (neoplasm$ or glioblastoma$ or cancer* or tumour$ or tumor$ or malignan$ or carcinoma$ or adenocarcinoma$ or oncolog$ or gliom$).ti,ab |
| 6 | 4 and 5 |
| 7 | (surviv* or mortality or death*).ti,ab,kw |
| 8 | 6 and 7 |
| 9 | (animal$ not human$).sh,hw. |
| 10 | conference abstract.pt |
| 11 | (editorial or comment* or letter or note or case study or case studies or case report).pt. or (editorial/ or letter/ or case study/ or case report/ or note/) or (case report* or case stud*).ti,ab |
| 12 | (infan* or child* or adolescen* or pediatr* or paediatr* or juvenile).ti,ab,kw. |
| 13 | or/9–12 |
| 14 | 8 not 13 |
| 15 | limit 14 to English language |
| 16 | limit 16 to yr="2011–Current" |

Reproduced from Bonomi PD et al. Mortality burden of pre-treatment weight loss in patients with non-small-cell lung cancer: A systematic literature review and meta-analysis. Journal of Cachexia, Sarcopenia and Muscle, 2024 Apr 22 [19] licensed under CC BY 4.0.

**Table S2.** PubMed search strategy.

| **Step** | **Search string** |
| --- | --- |
| 1 | "cachexia"[MeSH Terms] OR "cachexia"[All Fields] OR "cachexias"[All Fields] OR "sarcopenia"[MeSH Terms] OR "emaciation"[Title/Abstract] OR "wasting"[Title/Abstract] OR "weight loss"[Title/Abstract] OR "weight reduction"[Title/Abstract] OR "sarcopenia"[Title/Abstract] OR "sarcopaenia"[Title/Abstract] |
| 2 | "neoplasms"[MeSH Terms] OR "neoplasms"[All Fields] OR "neoplasm"[All Fields] OR "glioblastoma"[All Fields] OR "glioblastomas"[All Fields] OR "cancerous"[All Fields] OR "cancer"[All Fields] OR "cancers"[All Fields] OR "tumour"[All Fields] OR "tumor"[All Fields] OR "tumours"[All Fields] OR "tumors"[All Fields] OR "malign"[All Fields] OR "malignant"[All Fields] OR "malignancies"[All Fields] OR "malignancy"[All Fields] OR "carcinoma"[MeSH Terms] OR "carcinoma"[All Fields] OR "carcinomas"[All Fields] OR "carcinoma s"[All Fields] OR "adenocarcinoma"[MeSH Terms] OR "adenocarcinoma"[All Fields] OR "adenocarcinomas"[All Fields] OR "oncology"[All Fields] OR "glioma"[MeSH Terms] OR "glioma"[All Fields] OR "gliomas"[All Fields] |
| 3 | 1 and 2 |
| 4 | "survival"[Title/Abstract] OR "mortality"[Title/Abstract] OR "death"[Title/Abstract] |
| 5 | 3 and 4 |
| 6 | "editorial"[Publication Type] OR "comment"[Publication Type] OR "letter"[Publication Type] OR "case reports"[Publication Type] OR "animal*"[Title/Abstract] OR "in vitro"[Title/Abstract] OR "tissue*"[Title/Abstract] OR "murine"[Title/Abstract] OR "mouse"[Title/Abstract] OR "mice"[Title/Abstract] OR "swine*"[Title/Abstract] OR "pig"[Title/Abstract] OR "pigs"[Title/Abstract] OR "porcine"[Title/Abstract] OR "rat"[Title/Abstract] OR "rats"[Title/Abstract] OR "rodent*"[Title/Abstract] OR "monkey"[Title/Abstract] OR "monkeys"[Title/Abstract] OR "ape"[Title/Abstract] OR "apes"[Title/Abstract] OR "dog"[Title/Abstract] OR "dogs"[Title/Abstract] OR "canine*"[Title/Abstract] OR "cat"[Title/Abstract] OR "cats"[Title/Abstract] OR "feline*"[Title/Abstract] OR "cow"[Title/Abstract] OR "bovine"[Title/Abstract] OR "horse"[Title/Abstract] OR "equine"[Title/Abstract] OR "infant"[Title/Abstract] OR "infants"[Title/Abstract] OR "child"[Title/Abstract] OR "children"[Title/Abstract] OR "adolescent"[Title/Abstract] OR "adolescents"[Title/Abstract] OR "adolescence"[Title/Abstract] OR "pediatric"[Title/Abstract] OR "paediatric"[Title/Abstract] OR "juvenile"[Title/Abstract] |
| 7 | 5 not 6 |
| 8 | Limit to English language |
| 9 | Limit from 2011–2021 |

Reproduced from Bonomi PD et al. Mortality burden of pre-treatment weight loss in patients with non-small-cell lung cancer: A systematic literature review and meta-analysis. Journal of Cachexia, Sarcopenia and Muscle, 2024 Apr 22 [19] licensed under CC BY 4.0.

**Table S3.** Design and subject characteristics of colorectal cancer studies identified during the SLR (*n*=13)

| **Author; Year;  Country; Reference** | **Study design; Sample size; Study period; Follow-up** | **Setting; Study cohort(s); Age; % Female** | **BMI, kg/m^2^, mean (SD)^a^** | **Tumor stage; PS, n (%)** | **Definition of cachexia or weight loss** | **Prevalence of cachexia or weight loss, *n* (%)** |
| --- | --- | --- | --- | --- | --- | --- |
| Best et al.; 2021; US;  [29] | Retrospective; *N*=492 (3-mo FU: *n*=193; 6-mo FU: *n*=164; 12-mo FU: *n*=135); Jan 2014 to Aug 2018; FU: Mean (SD): 89 days (20) | Institution; Age, y  3-mo FU: Mean (SD): 58.9 (13.1); Median (IQR): 59 (50–68)  6-mo FU: Mean (SD): 59.3 (13.5); Median (IQR): 59 (50–68)  12-mo FU: Mean (SD): 58.0 (13.1); Median (IQR): 59 (50–68)  % Female:  3-mo FU: 46.6%  6-mo FU: 45.7%  12-mo FU: 48.1% | 3-mo FU:  Mean (SD): 27.5 (5.4); Median (IQR): 27 (24–30)  6-mo FU:  Mean (SD): 27.5 (5.5); Median (IQR): 27 (24–30)  12-mo FU:  Mean (SD): 27.6 (5.5); Median (IQR): 27 (24–31) | NR;  NR | IC definition:  Cancer cachexia is a multifactorial syndrome that has traditionally been defined using WL (e.g., >5% WL over 6 mo). WL assessed at 3, 6, and 12 mo following cancer diagnosis | Pts with WL >5% at 3 mo post diagnosis: 43 (22) |
| Gannavarapu et al.;  2018; NR (US assumed);  [20] | Retrospective;  Colorectal cohort: *N*=623;  Jan 1, 2006 to Dec 31, 2013;  FU: NR | NR;  NR;  NR | NR | NR;  NR | IC definition:  Overt WL was defined as UWL >5% within 6 mo preceding cancer diagnosis in pts with BMI >20 kg/m^2^ or UWL >2% in pts with BMI <20 kg/m^2^ | Cachexia (overt WL):  172 (27.6)  Minimal WL: 25 (4.0) |
| Guercio et al.; 2020;  US  [30] | Prospective; *N*=2323:  BMI, kg/m^2^ <21: *n*=248  BMI, kg/m^2^ 21–24.9: *n*=532  BMI, kg/m^2^ 25–29.9: *n*=823  BMI, kg/m^2^ 30–34.9: *n*=463  BMI, kg/m^2^ ≥35: *n*=257  Study period: NR  FU: Median: 5.98 y | NR;  Age, y, median (IQR):  BMI <21: 57.7 (49.3–66.4)  BMI 21–24.9: 59.3 (51.5–68.5)  BMI 25–29.9: 60.2 (52.1–68.7)  BMI 30–34.9: 58.5 (51.2–66.8)  BMI ≥35: 57.6 (49.3–63.8)  % Female:  BMI <21: 51.6%  BMI 21–24.9: 42.2%  BMI 25–29.9: 36.3%  BMI 30–34.9: 40.1%  BMI ≥35: 51% | Median (IQR): 19.5 (18.3–20.2) | Tumor grade: NR;  ECOG PS:  BMI <21:  0: 119 (48.0)  1: 127 (51.2)  2: 2 (0.8)  BMI 21–24.9:  0: 291 (54.7)  1: 241 (45.3)  2: NR  BMI 25–29.9:  0: 508 (61.7)  1: 315 (38.3)  2: NR  BMI 30–34.9:  0: 295 (63.7)  1: 168 (36.3)  2: NR  BMI ≥35:  0: 146 (56.8)  1: 110 (42.8)  2: 1 (0.4) | Broad definition:  % change in weight (loss >15%, loss 10.1%–15%, loss 5%–10%, stable ±4.9% [reference group], and gain ≥5%) at study entry (before study treatment) | Pts with WL  BMI <21:  WL ≥5%: 96 (38.7)  Missing: 123 (49.6)  BMI 21–24.9:  WL ≥5%: 202 (38.0)  Missing: 230 (43.2)  BMI 25–29.9:  WL ≥5%: 322 (39.1)  Missing: 325 (39.5)  BMI 30–34.9:  WL ≥5%: 159 (34.3)  Missing: 188 (40.6)  BMI ≥35:  WL ≥5%: 89 (34.6)  Missing: 104 (40.5) |
| Islam et al.; 2020;  US;  [31] | Retrospective; Total cohort: *N*=149;  2002–2009;  FU: Median: 19.4 mo; Median (range): 581 days (37–2294) | Oncology outpatient;  Age, y:  Mean (SD): 63.5 (11.0)  Median (range): 63.9 (28.0–92.0);  % Female: 48% | 27.8 (5.5) | Tumor stage (T3–4):  Yes: 130 (87)  No: 19 (13)  Tumor grade (3–4):  Yes: 115 (80)  No: 28 (20)  Lymph node (N1–2):  Yes: 99 (66)  No: 50 (34)  Metastasis (M1):  Yes: 102 (68)  No: 47 (32); | Broad definition:  Unexplained WL >10%; Unable to determine if WL is before diagnosis, during treatment, or both | Pts with unexplained WL >10%: 42 (32.1)  *Denominator NR; assumed to be 131 based on pts with WL data* |
| Karabulut et al.;  2021;  Turkey;  [39] | Prospective;  Total cohort: *N*=137;  Feb 2018 to Mar 2019;  FU, median (95% CI): 4 mo (1–13) | Oncology outpatient;  Age, y, median (range): 62 (18–83);  % Female: 61% | BMI, *n* (%)  Underweight (<18.5): 5 (0.4)  Normal weight (18.5–25): 60 (44)  Overweight (25–30): 36 (26)  Obesity (>30): 32 (23) | Metastasis site,  Liver: 105 (77)  Lung: 42 (31)  Number of metastases,  Single site: 66 (48)  Multiple sites: 77 (52);  WHO PS,  0: 47 (34)  1: 90 (64) | Broad definition: >10% WL, assessed before the first CT, and after first and second CT during 2 cycles of CT every 15 days | Pts with WL,  >10% WL: 34 (25)  <10% WL: 55 (40)  Former body WL% median (range): 8.5 (1.3–27.7) |
| Kocarnik et al.; 2017;  US, Australia, Canada;  [32] | Prospective;  Total cohort: *N*=2,049  Lost weight (>5%): *n*=386  Maintained weight (≤5% change): *n*=985  Gained weight (>5%): *n*=678;  1997–2008;  FU, median (range): 5.1 y (0.3–9.9) | NR, oncology outpatient assumed;  Total cohort: 54.8 (11.1) y;  % Female: 50% | Total cohort:  Baseline: 27.4 (5.4)  FU: 28.0 (5.8)  Change in BMI: 0.6 (3.4) | AJCC stage of disease at diagnosis,  Total cohort:  I: 571 (28)  II: 565 (28)  III: 415 (20)  IV: 130 (6)  Missing: 368 (18);  PS, NR | Broad definition:  WL >5% in the 5 years following diagnosis | Pts with WL >5%: 386 (19) |
| Lee et al.; 2020; International; [33] | Retrospective;  Total cohort: *N*=3,449;  Dec 2004 to Jun 2007;  FU, mean: 37.8 mo | NR;  Total cohort:  Age, y, *n* (%)  <65: 2,456 (71.2)  ≥65: 993 (28.8);  % Female: 45.8% | BMI, *n* (%)  Underweight, <18.5: 93 (2.7)  Normal weight, 18.5–24.9: 1643 (47.6)  Overweight, 25–29.9: 1239 (35.9)  Obese, ≥30: 474 (13.7) | Tumor grade/stage,  Total cohort:  Stage II (high-risk): 577 (16.7)  Stage III, N1: 1750 (50.7)  Stage III, N2: 1122 (32.5);  ECOG PS,  Total cohort:  0: 2959 (85.8)  1: 487 (14.1)  Missing: 3 (0.1) | Broad definition: WL ≥5% during first 6 mo of adjuvant CT (following curative surgery) | Pts (data available for *N*=2455) with WL,  WL 5–9.9%: 175 (7.1)  WL ≥10%: 77 (3.1) |
| Liu et al.; 2021; Germany;  [34] | Retrospective;  Total cohort (pts with available body weight data): *N*=326; Study period: NR; FU: NR | Institution;  Total cohort:  Age, y, *n* (%)  <65: 162 (49.7)  ≥65: 164 (50.3)  % Female: 27.6% | BMI, *n* (%),  Total cohort:  BMI <30: 266 (81.8)  BMI ≥30: 59 (18.2)  Missing: 1 (0.3) | Total cohort,  Number of metastatic sites:  <2: 139 (42.8)  ≥2: 186 (57.2)  Missing: 1 (0.3);  ECOG PS, Total cohort:  0: 180 (55.2)  1 and 2: 146 (44.8) | Broad definition:  EWL as body WL of ≥5% after 3 mo of treatment | Pts with WL ≥5%: 47 (14.4) |
| Meyerhardt et al.;  2017; US;  [35] | Retrospective;  Large loss (≥10%): *n*=239  Modest loss (5–9.9%): *n*=309  Stable (−4.9 to 4.9%): *n*=1460  Modest gain (5–9.9%): *n*=453  Large gain (≥10%): *n*=320  Overall: *N*=2781;  2006–2011  FU, median (range):  4.2 y (0.1–8.1) | NR (oncology outpatient assumed);  Overall: median (IQR) age at diagnosis: 63 y (NR)  % Female: 50.4% | Overall, BMI at baseline (%):  <25: 32.4  25 ≤ 30: 36.2  30 ≤ 35: 19.7  ≥35: 11.7  BMI at FU (%):  <25: 31.1  25 ≤ 30: 34.9  30 ≤ 35: 21.5  ≥35: 12.5 | Tumor stage (%),  Overall:  I: 28.7  II: 32.1  III: 39.2;  PS, NR | Broad definition:  moderate WL 5–9.9% or large WL ≥10% assessed at 3 mo and 18 mo following diagnosis | Pts with WL,  Large loss (≥10%): 239 (8.6)  Modest loss (5–9.9%): 309 (11.1) |
| Shibata et al.; 2020;  Japan;  [16] | Retrospective  Total cohort: *N*=150;  Feb 1, 2010, to Aug 31, 2016;  FU: NR | Oncology outpatient;  Age, y, median (range): 65.0 (29–85)  % Female: 40% | Median (range): 21.72 (14.8–32.5) | UICC stage,  III: 1 (0.7)  IV: 149 (99.3);  ECOG PS,  0: 75 (50.0)  1: 65 (43.3)  2: 9 (6.0)  3: 1 (0.7) | IC definition:  Cancer cachexia as either (1) WL of >5% in the past 6 mo or (2) WL of >2% in the past 6 mo with BMI of <20 kg/m^2^ after start of 1L CT | Cachexia prevalence  42.7% within 12 wk, 50.7% at 24 wk, 65.3% at 48 wk and reached 91.3% over the whole study period  91 pts (60.7%) during 1L CT |
| Silva et al.; 2020; Brazil;  [36] | Retrospective;  Total cohort: *N*=148;  Jan 2008 to Dec 2012;  FU: 5 y | Institution;  Age, y, mean (SD): 62.1 (12.8);  % Female: 48% | BMI classification, *n* (%)  Undernourished/low weight: 28 (18.9)  Eutrophy: 56 (37.9)  Overweight/obesity: 64 (43.2) | Tumor staging,  I/II: 13 (8.8)  III/IV: 106 (71.6)  Uninformed: 29 (19.6);  Presence of metastasis,  Yes: 35 (23.6)  No: 113 (76.4);  PS, NR | Broad definition:  WL significance defined according to Blackburn et al. (1977)^b^ which considers periods and % of WL, assessed during first hospital stay prior to treatment^b^ | WL classification,  Significant loss: 33 (22.3)  Severe loss: 40 (27.0) |
| Vergidis et al.; 2016; Canada;  [37] | Retrospective;  Total cohort: *N*=539  Weight change <5%: *n*=175  Weight change ≥5%: *n*=364  WL <5%: *n*=471  WL ≥5%: *n*=68  WG <5%: 175  WG ≥5%: 364  Weight change <10%: *n*=404  Weight change ≥10%: *n*=135  WL <10%: 516  WL ≥10%: 23  WG <10%: 440  WG ≥10%: 99;  2008–2010;  FU: 3–5 y | Oncology outpatient;  Age, y,  Total cohort:  Median (range): 69 (26–94)  WL <5%:  ≤70, %: 60.9  >70, %: 39.1  WL ≥5%:  ≤70, %: 66.2  >70, %: 33.8  % Female,  Total cohort: 48% Female  WL <5%: 52%  WL ≥5%: 34% | %,  Total cohort: NR  WL <5%:  <25: 46.9  25–30: 35.7  >30: 17.4  WL ≥5%:  <25: 29.8  25–30: 44.8  >30: 24.4 | Total cohort,  Stage III: 539 (100)  ECOG PS 0–1: 53%  WL <5%,  Surgical T stage, (%)  1: 2.4  2: 9.6  3: 68.7  4: 19.3  Surgical N stage, (%)  0: 0  1: 66.1  2: 33.9  WL ≥5%,  Surgical T stage, (%)  1: 2.9  2: 11.8  3: 64.7  4: 20.6  Surgical N stage, (%)  0: 1.5  1: 63.2  2: 35.3 | Broad definition:  WL ≥5% or WL ≥10% between baseline and during adjuvant CT | Pts with WL,  Total cohort:  WL ≥5%: 68 (12.6)  WL ≥10%: 23 (4.3) |
| Walter et al.; 2016; Germany;  [38] | Retrospective;  Total cohort: *N*=3130  BMI <20 kg/m^2^: 140  BMI 20 to <25 kg/m^2^: 1062  BMI 25 to <30 kg/m^2^: 1321  BMI ≥30 kg/m^2^: 593;  2003–2010;  FU, median: 4.9 y | Inpatient;  Total cohort  Age, median (range):  69 (30–96) y;  % Female: 40.7% | Total cohort, *n* (%):  <20: 140 (4.5)  20 to <25: 1062 (33.9)  25 to <30: 1321 (42.2)  ≥30: 593 (18.9) | Total cohort:  Tumor stage,  I: 695 (22.2)  II: 963 (30.8)  III: 1,023 (32.7)  IV: 449 (14.3);  PS, NR | Broad definition:  Martin et al., 2015^c^ grading criteria, assessed prior to diagnosis:  BMI 20 to <25 and WL <2.5%  BMI ≥28 and WL 2.5 to <6%  BMI 20 to <28 and WL 2.5 to <6% or  BMI ≥28 and WL 6 to <11%  BMI <20 and WL <6% or  BMI 20 to <28 and WL 6 to <11%, or  BMI ≥22 and WL 11 to <15% BMI ≥28 and WL ≥15%,  BMI <20 and WL ≥6%, or  BMI 20 to <22 and WL ≥11% or  BMI 22 to <28 and WL ≥15%  BMI ≥25 and WL <2.5% ( reference group) | WL grades (proposed by Martin et al., 2015^c^), *n*,  Grade 0, BMI ≥25 and WL <2.5%: 520  Grade 1, BMI 20 to <25 and WL <2.5% or BMI ≥28 and WL 2.5 to <6%: 403  Grade 2, BMI 20 to <28 and WL 2.5 to <6% or BMI ≥28 and WL 6 to <11%: 451  Grade 3, BMI <20 and WL <6% or BMI 20 to <28 and WL 6 to <11%: 591  Grade 4, BMI <20 and WL ≥6%, or BMI 20 to <22 and WL ≥11% or BMI 22 to <28 and WL ≥15%: 284 |

^a^Unless otherwise stated.

^b^Blackburn GL, Bistrian BR, Maini BS, Schlamm HT, Smith MF. Nutritional and metabolic assessment of the hospitalized patient. JPEN J Parenter Enteral Nutr. 1977;1:11-22.

^c^Martin L, Senesse P, Gioulbasanis I, Antoun S, Bozzetti F, Deans C, et al. Diagnostic criteria for the classification of cancer-associated weight loss. J Clin Oncol. 2015;33:90-9.

1L, first line; AJCC, American Joint Committee on Cancer; BMI, body mass index; CI, confidence interval; CT, chemotherapy; ECOG, Eastern Cooperative Oncology Group; EWL, early weight loss; FU, follow-up; IC, international consensus; IQR, interquartile range; mo, months; NR, not reported; PS, performance status; pts, patients; SD, standard deviation; T/N/M, tumor/node/metastasis; UICC, Union for International Cancer Control; UWL, unintentional weight loss; WHO, World Health Organization; WG, weight gain; wk, weeks; WL, weight loss; y, years

**Table S4.** Design and subject characteristics of pancreatic cancer studies identified during the SLR (*n*=13)

| **Author; Year;  Country; Reference** | **Study design; Sample size; Study period; Follow-up** | **Setting; Study cohort(s); Age; % Female** | **BMI, kg/m^2^, mean (SD)^a^** | **Tumor stage; PS, *n* (%)** | **Definition of cachexia or weight loss** | **Prevalence of cachexia or weight loss, *n* (%)** |
| --- | --- | --- | --- | --- | --- | --- |
| Arthur et al.;  2016;  US;  [47] | Cross-sectional;  Pancreatic cohort: *N*=17,237  Pts with cachexia: *n*=1148  Pts without cachexia: *n*=16,089;  Jan 2009 to Dec 2009;  FU: NR | Inpatient;  Pancreatic cohort:  Pts with cachexia: 67.84 (NR) y  Pts without cachexia: 68.01 (NR) y  % Female:  Pts with cachexia: 47.3%  Pts without cachexia: 49.2% | NR | NR;  NR | Broad definition:  ICD-9 codes for cachexia (ICD-9 799.4), any diagnosis for loss of weight (ICD-9 783.21), anorexia (ICD-9 783.0), or being underweight (ICD-9 783.22) (Fox et al, 2009 [48]) at hospital admission  Unable to determine if WL is before diagnosis, during treatment, or both | Pancreatic cancer cohort:  Cachexia prevalence: 1148 (6.7) |
| Carnie et al.;  2020;  UK;  [40] | Retrospective;  Total cohort: *N*=255  Pts with WL <5%, wk 4: *n*=196  Pts with WL ≥5%, wk 4: *n*=59;  Jan 2015 to Jan 2019;  FU, Median: 8.5 mo | Oncology outpatient;  Total cohort:  Median (range): 67 (25–85) y  % Female: 45.5%  Pts with WL <5%, week 4 cohort  Median (Range): 67 (25–85) y  % Female: 48.9%  Pts with WL ≥5%, wk 4 cohort  Median (range): 65 (40–83) y  % Female: 33.9% | NR | Stage,  Total cohort:  Localized/locally advanced: 121 (47.5)  Metastatic: 134 (52.5);  Pts with WL <5%:  Localized/locally advanced: 95 (48.5)  Metastatic: 101 (50.5)  Pts with WL ≥5%:  Localized/locally advanced: 26 (44.1)  Metastatic: 33 (55)  ECOG PS at cycle 1 day 1 of CT:  Total cohort:  0–1: 220 (86.3)  2+: 34 (13.7);  Pts with WL <5%:  0–1: 167 (85.1)  2+: 29 (14.9);  Pts with WL ≥5%:  0–1: 54 (91.5)  2+: 5 (8.5) | Broad definition:  ≥5% loss of body WL during first 4 wk of CT | Pts with WL ≥5% at wk 4: 59 (23.1) |
| Domínguez-Muñoz et al.;  2018;  Spain;  [45] | Retrospective;  Total cohort *N*=160  Group 1 (pts received palliative CT only) cohort: *n*=86 (53.75%)  Group 2 (palliative CT + pancreatic enzyme replacement therapy) cohort: *n*=74 (46.25%);  Jan 2011 to Oct 2016;  FU: NR | Institution;  Group 1 (palliative CT only):  Median (range): 71.5 (43–100) y  % Female: 39.5%  Group 2 (palliative CT + pancreatic enzyme replacement therapy):  Median (range): 69.5 (28–90) y  % Female: 45.9% | NR | Group 1 (palliative CT only):  Tumor stage (locally advanced/metastatic): 16/70  Tumor location (head/body/tail): 46/33/7  Tumor size, mean ± SD (cm): 4.5 ± 1.5;  PS: NR  Group 2 (palliative CT + pancreatic enzyme replacement therapy):  Tumor stage (locally advanced/metastatic): 18/56, *p*=0.440  Tumor location (head/body/tail): 47/21/6, *p*=0.396  Tumor size, mean ± SD (cm): 4.1 ± 1.5, *p*=0.828;  PS: NR | Broad definition: Significant WL (>10%) of body weight over <6 mo before diagnosis | Group 1 (palliative CT only):  WL >10% at diagnosis: 47 (54.7)  Group 2 (palliative CT + pancreatic enzyme replacement therapy):  WL >10% at diagnosis: 48 (64.9) |
| Duconseil et al.  2019;  France;  [41] | Prospective;  Total cohort: *N*=454;  Jan 2005 to Dec 2017  FU, mean: 22 mo | Institution;  Total cohort, mean (SD): 65 (10) y;  Sex ratio (F/M): 0.96 | Mean BMI (SD): 23.6 (4.16);  BMI kg/m^2^, *n* (%)  <18.5 (%): 37 (8.2)  18.5–24.9 (%:) 282 (62.1)  25–29.9 (%): 105 (23.1)  ≥30 (%): 30 (6.6) | Tumor staging  Borderline: 336 (74)  Locally advanced: 118 (26);  PS at diagnosis  0–1 (%): 397 (87.4)  2–3 (%): 58 (12.6) | Broad definition: WL >5% at diagnosis when compared with the usual weight within the last 6 mo prior to diagnosis or first disease symptoms  Continuous WL, defined as a decrease in weight between initial and restaging evaluation, in patients who already had WL >5% at diagnosis | Pts with WL >5% at diagnosis: 294 (64.8)  Continuous WL at restaging (during induction treatment): 60 (13.2) |
| Gannavarapu et al.  2018;  NR (US assumed); [20] | Retrospective  Pancreatic cohort: *N*=267  WL >5% (overt WL): *n*=142  WL <5% (minimal WL): *n*=15  No WL: 110;  Jan 2006 to Dec 2013;  FU: NR | NR;  NR;  NR | NR | NR;  NR | IC definition:  Overt WL was defined as UWL >5% within 6 mo before cancer diagnosis in pts with BMI >20 kg/m^2^ or UWL >2% in pts with BMI <20 kg/m^2^ | Pancreatic cohort:  Cachexia prevalence (overt WL): 142 (53.2),  Minimal WL: 15 (5.6) |
| Hendifar et al.;  2018;  US;  [17] | Retrospective;  Total cohort: *N*=977  Cachexia cohort: *n*=611  No cachexia cohort: *n*=366;  2006 to 2014;  FU: NR | Institution;  Cachexia cohort:  Mean (SD): 69.9 (10.1) y  % Female: 47.1%;  No cachexia cohort:  Mean (SD): 70.1 (11.3) y  % Female: 50.3% | BMI kg/m^2^, *n* (%)  Cachexia cohort:  <18.5: 9 (1.5)  18.5–24.9: 153 (25.0)  25.0–29.9: 220 (36.0)  ≥30.0: 229 (37.5)  No cachexia cohort:  <18.5: 11 (3.0)  18.5–24.9: 118 (32.2)  25.0–29.9: 131 (35.8)  ≥30.0: 106 (29.0) | Cachexia cohort:  Stage 1: 52 (8.5)  Stage 2: 174 (28.5)  Stage 3: 51 (8.3)  Stage 4: 334 (54.7)  PS: NR  No cachexia cohort:  Stage 1: 26 (7.1)  Stage 2: 119 (32.5)  Stage 3: 22 (6.0)  Stage 4: 199 (54.4);  PS: NR | IC definition: Cancer cachexia at diagnosis was defined as WL >5% over the 6 mo of evaluation, or WL >2% in individuals already showing depletion according to current body weight and height (BMI <20 kg/m^2^ or sarcopenia) | Cachexia prevalence: 611/977 (62.5) |
| Hue et al.;  2021;  US;  [46] | Retrospective;  Overall (PDAC): *N*=390;  2009–2019;  FU, median (range):  PDAC, 365 days cohort: 170 days (7–365)  PDAC, 180 days cohort: 111 days (7–180) | Institution  Overall (PDAC), Median (range): 71 (35–98) y;  % Female: 56.7% | NR | Overall (PDAC)  Stage at diagnosis:  I: 22 (5.6%)  II: 104 (26.7%)  III: 52 (13.3%)  IV: 198 (50.8%)  Incomplete: 14 (3.6%);  PS: NR | Broad definition:  WL was grouped as 5% to <10% WL, 10% to <15% WL, and ≥15% WL in either 180 or 365 days before diagnosis | 365 days preceding (cases *n*=390):  WL 5% to 10%: 148 (37.9)  WL ≥10%: 144 (36.9)  180 days preceding (cases, *n*=361) :  WL 5% to 10%: 146 (40.4)  WL ≥10%: 96 (26.7) |
| Latenstein et al.;  2020;  Netherlands;  [18] | Prospective;  Total cohort: *N*=202  Palliative care (non-surgical) pts who completed questionnaire at baseline: *n*=70  Jan 2015 to Feb 2018;  FU: 22 mo | Institution  Total cohort:  Median (IQR): 68 (62–73) y  % Female: 47%  Palliative care cohort:  Median (IQR): 65: (60-70) y;  % Female: 50% | Palliative care cohort:  Self-reported BMI, median (IQR): 25 (22–26)  BMI kg/m^2^, *n* (%)  <18.5: 1 (1%)  18.5–25: 40 (57%)  ≥25: 29 (41%) | Palliative care cohort,  Tumor grade:  I: 1 (1%)  II: 11 (26%)  III: 19 (27%)  IV: 34 (49%);  WHO PS:  0 or 1: 52 (49)  ≥2: 7 (10)  Missing: 11 (16) | IC definition: Cachexia is defined as WL >5%, or WL >2% in individuals with BMI <20 kg/m^2^ or low skeletal muscle mass (sarcopenia) during the past 6 mo, measured at baseline (cannot determine if cachexia was pre-diagnosis or during treatment) | Total cohort:  Cachexia prevalence: 144 (71)  WL ≥10% at baseline: 81 (40)  Palliative care cohort:  Cachexia prevalence: 54 (77)  WL ≥10% at baseline: 31 (44) |
| Mitsunaga et al.;  2020;  Japan;  [42] | Retrospective;  Total cohort: *N*=150  Jun 2008 to Mar 2017;  FU: NR | Oncology outpatient;  Total cohort:  Median (range): 65 (35–83) y  % Female: 41.3%; | Total cohort:  Median (range): 21.7 (13.8–33.3) | UICC stage  Total cohort:  III: 44 (29.3)  IV: 106 (70.7)  ECOG PS:  Total cohort:  0: 106 (70.7)  1: 44 (29.3) | IC definition**:**  Cancer cachexia as either WL >5% or WL >2% with BMI <20 kg/m^2^ Base cachexia defined as WL within 6 mo before start of CT  FU cachexia was assessed at 1–12, 13–24, 25–36, 37–48, and >48 wk from CT initiation | Total cohort, cancer cachexia at start of 1L CT (base cachexia): 75 (50.0) |
| Naumann et al.;  2019;  Germany;  [27] | Retrospective;  Total cohort: *N*=141;  2007–2014;  FU: NR | Institution;  Mean (SD): 63.6 (9.2) y;  % Female: 45.4% | NR | Tumor stage:  I: 0 (0)  II: 0 (0)  III: 141 (100)  IV: 0 (0);  ECOG PS:  0: 72 (51.1)  1: 58 (41.1)  2: 11 (7.8)  3: 0 (0) | IC definition:  Defined as WL >5% of usual body weight at the time of cancer diagnosis; also assessed after CRT at first FU | WL >5%, before CRT (baseline): 83 (58.9)  Cachexia by definition: 89 (63.1)  WL >5%, after CRT: 120 (85.1) |
| Naumann et al.;  2019;  Germany;  [28] | Retrospective;  Total cohort: *N*=147;  2007 to 2014;  FU: NR | Institution;  Total cohort, mean (SD): 63.6 (9.0) y;  % Female: 46.3% | Total cohort, mean (SD; range): 24.1 (±3.8; 17.2–46.7)  BMI WHO class distribution, *n* (%) Underweight (BMI <18.5): 3 (2.1)  Normal (18.5 ≤ BMI <25): 89 (60.5)  Pre-obese (25 ≤ BMI <30): 47 (32.0)  Obesity (≥30): 8 (5.4) | Total cohort,  Tumor stage:  I: 0 (0%)  II: 0 (0%)  III: 147 (100%)  IV: 0 (0%);  ECOG PS,  0: 75 (51.0)  1: 60 (40.8)  2: 12 (8.2)  3: 0 (0) | IC definition:  Cancer cachexia was defined as either a WL >5% or a depletion in muscle mass below sex-specific cut-offs in combination with persistent WL ≥2% during CRT | Total cohort:  WL >5%: 71 (48.3) |
| Nemer et al.;  2017;  US;  [44] | Retrospective;  Total cohort: *N*=123  Jan 2014 to Dec 2014;  FU: NR | Oncology outpatient;  Total cohort, mean (SD): 65.9 (11.3) y;  % Female: 48.8% | Total cohort:  BMI at diagnosis: 28.1 (7.0)  Usual BMI: 31.2 (7.9) | Total cohort,  Cancer stage:  Early (stages I–II): 64 (52.0)  Late (stages III–IV): 59 (48.0)  PS: NR | IC definition:  Cachexia was defined as WL of >5% of the usual body weight at cancer diagnosis | Total cohort:  WL >5%: 88 (71.5)  WL >10%: 48 (39.0) |
| Ramsey et al.;  2019;  US;  [43] | Retrospective;  Total cohort: *N*=136  Cohort B (clinical trial pts who underwent CT): *n*=56;  Jan 2011 to Jul 2016;  FU: NR | Institution;  Total cohort, mean (SD): 65.5 (10.3) y  Cohort B (CT), mean (SD): 62.6 (10.0) y  Total cohort: 45.6% Female  Cohort B (CT): 37.5% Female | Total cohort:  Usual BMI: 30.6 (6.5)  BMI at diagnosis: 27.7 (5.4)  Cohort B (chemo)  Usual BMI: 30.4 (6.0)  BMI at diagnosis: 27.7 (4.4) | AJCC cancer stage,  Total cohort:  IA: 6 (4.4)  IIA: 7 (5.2)  IIB: 49 (36.0)  III: 10 (7.4)  IV: 64 (47.1)  Cohort B (CT):  IA: 0 (0.0)  IIA: 0 (0.0)  IIB: 1 (1.8)  III: 0 (0.0)  IV: 55 (98.2)  ECOG PS at diagnosis,  Total cohort:  0: 51 (38.9)  1: 70 (53.4)  2: 8 (6.1)  3: 1 (0.8)  4: 1 (0.8)  Cohort B (CT):  0: 36 (64.3)  1: 19 (33.9)  2: 1 (1.8)  3: 0 (0.0)  4: 0 (0.0) | IC definition:  Cachexia was defined as WL >5% of usual body weight at the time of cancer diagnosis | Total cohort:  WL >5%: 70 (56.9)  WL >10%: 38 (30.9);  Cohort B (chemo):  WL >5%: 27 (50.9)  WL >10%: 15 (28.3) |

^a^Unless otherwise stated.

1L, first line; AJCC, American Joint Committee on Cancer; BMI, body mass index; CRT, chemoradiotherapy; CT, chemotherapy; ECOG, Eastern Cooperative Oncology Group; F, female; FU, follow-up; IC, international consensus; ICD-9, International Classification of Diseases, 9th ed; IQR, interquartile range; M, male; mo, months; NR, not reported; PDAC, pancreatic ductal adenocarcinoma; PS, performance status; pts, patients; SD, standard deviation; UWL, unintentional weight loss; WHO, World Health Organization; wk, weeks; WL, weight loss; y, years.

**Table S5.** Quality assessment of observational studies using the Newcastle–Ottawa Scale

| **Author; Year; Reference** | **Selection** | | | | **Comparability** | **Outcome** | | | **Total** |
| --- | --- | --- | --- | --- | --- | --- | --- | --- | --- |
|  | Representativeness of the exposed cohort | Selection of the non-exposed cohort | Ascertainment of exposure | Demonstration that outcome of interest was not present at start of study | Comparability of cohorts based on the design or analysis | Assessment of outcome | Was follow-up long enough for outcomes to occur? | Adequacy of follow-up of cohorts | Total score (out of 9) |
| **Colorectal cancer studies** |  |  |  |  |  |  |  |  |  |
| Best et al.; 2021; [29] | 0 | 1 | 1 | 1 | 2 | 1 | 1 | 1 | 8 |
| Gannavarapu et al.; 2018; [20] | 0 | 1 | 1 | 1 | 2 | 1 | 1 | 1 | 8 |
| Guercio et al.; 2020; [30] | 1 | 1 | 1 | 1 | 2 | 1 | 1 | 1 | 9 |
| Islam et al.; 2020; [31] | 0 | 1 | 1 | 1 | 2 | 1 | 1 | 1 | 8 |
| Karabulut et al.; 2021; [39] | 1 | 1 | 1 | 1 | 0 | 1 | 1 | 1 | 7 |
| Kocarnik et al.; 2017; [32] | 1 | 1 | 0 | 1 | 2 | 1 | 1 | 1 | 8 |
| Lee et al.; 2020; [33] | 1 | 1 | 1 | 1 | 2 | 1 | 1 | 1 | 9 |
| Liu et al.; 2021; [34] | 1 | 1 | 1 | 1 | 2 | 1 | 1 | 1 | 9 |
| Meyerhardt et al.; 2017; [35] | 1 | 1 | 1 | 1 | 2 | 1 | 1 | 1 | 9 |
| Shibata et al.; 2020; [16] | 1 | 1 | 1 | 1 | 2 | 1 | 1 | 1 | 9 |
| Silva et al.; 2020; [36] | 0 | 1 | 1 | 1 | 2 | 1 | 1 | 1 | 8 |
| Vergidis et al.; 2016; [37] | 1 | 1 | 0 | 1 | 2 | 1 | 1 | 1 | 8 |
| Walter et al.; 2016; [38] | 1 | 1 | 1 | 1 | 2 | 1 | 1 | 1 | 9 |
| **Pancreatic cancer studies** |  |  |  |  |  |  |  |  |  |
| Carnie et al.; 2020; [40] | 0 | 1 | 1 | 1 | 2 | 1 | 1 | 1 | 8 |
| Domínguez-Muñoz et al.; 2018; [45] | 0 | 1 | 1 | 1 | 1 | 1 | 1 | 1 | 7 |
| Duconseil et al.; 2019; [41] | 0 | 1 | 1 | 1 | 2 | 1 | 1 | 1 | 8 |
| Gannavarapu et al.; 2018; [20] | 0 | 1 | 1 | 1 | 2 | 1 | 1 | 1 | 8 |
| Hendifar et al.; 2018; [17] | 1 | 1 | 1 | 1 | 2 | 1 | 1 | 1 | 9 |
| Hue et al.; 2021; [46] | 0 | 0 | 1 | 1 | 2 | 1 | 1 | 1 | 7 |
| Latenstein et al.; 2020; [18] | 1 | 0 | 1 | 1 | 2 | 1 | 1 | 1 | 8 |
| Mitsunaga et al.; 2020; [42] | 0 | 1 | 1 | 1 | 2 | 1 | 1 | 1 | 8 |
| Naumann et al.; 2019; [27] | 0 | 1 | 1 | 1 | 2 | 1 | 1 | 1 | 8 |
| Naumann et al.; 2019; [28] | 0 | 1 | 1 | 1 | 2 | 1 | 1 | 1 | 8 |
| Nemer et al.; 2017; [44] | 0 | 1 | 1 | 1 | 2 | 1 | 1 | 1 | 8 |
| Ramsey et al.; 2019; [43] | 1 | 1 | 1 | 1 | 2 | 1 | 1 | 1 | 9 |

Information on the NOS is available in Wells, GA et al. [24]. NOS score: ≥7, good; 4–6, fair; 0–3, poor.

NOS, Newcastle–Ottawa Scale.

**Table S6.** Quality assessment of cross-sectional studies using the modified Newcastle–Ottawa Scale

| **Author; Year; Reference** | **Selection** | | | | **Comparability** | **Outcome** | | **Total** |
| --- | --- | --- | --- | --- | --- | --- | --- | --- |
|  | Representativeness of the sample | Sample size | Respondents / non-respondents | Ascertainment of exposure | Comparability of different outcome groups based on the design or analysis | Assessment of outcome | Statistical test | Total score (out of 9) |
| Arthur et al.; 2016 [47] | 1 | 1 | 1 | 1 | 2 | 1 | 1 | 8 |

Information on the modified Newcastle–Ottawa Scale (NOS) is available in Herzog R, et al. [25]. Modified NOS score: ≥7, good; 4–6, fair; 0–3, poor.
